# Endosialin expression in relation to clinicopathological and biological variables in rectal cancers with a Swedish clinical trial of preoperative radiotherapy

**DOI:** 10.1186/1471-2407-11-89

**Published:** 2011-03-01

**Authors:** Zhi-Yong Zhang, Hong Zhang, Gunnar Adell, Xiao-Feng Sun

**Affiliations:** 1 Department of Oncology, Institute of Clinical and Experimental Medicine, Linköping University, Linköping, S-581 85, Sweden; 2 Department of Pathology, Tangshan Gongren Hospital, Tangshan, PR China; 3 Laboratory Centre, First Hospital of Hebei Medical University, Shijiazhuang, PR China; 4 Division of Biomedicine, School of Life Science, University of Skövde, Skövde, Sweden; 5 Department of Oncology, Karolinska University Hospital, Stockholm, Sweden

## Abstract

**Background:**

The importance of changes in tumour-associated stroma for tumour initiation and progression has been established. Endosialin is expressed in fibroblasts and pericytes of blood vessels in several types of tumours, and is involved in the progression of colorectal cancer. In order to see whether endosialin was related to radiotherapy (RT) response, and clinicopathological and biological variables, we investigated endosialin expression in rectal cancers from the patients who participated in a Swedish clinical trial of preoperative RT.

**Methods:**

Endosialin was immunohistochemically examined in normal mucosa, including distant (*n *= 72) and adjacent (*n *= 112) normal mucosa, and primary tumours (*n *= 135). Seventy-three of 135 patients received surgery alone and 62 received additional preoperative RT.

**Results:**

Endosialin expression in the stroma increased from normal mucosa to tumour (*p *< 0.0001) both in RT and non-RT group. In the RT group, endosialin expression in the stroma was positively associated with expression of cyclooxygenase-2 (Cox-2) (*p *= 0.03), p73 (*p *= 0.01) and phosphates of regenerating liver (PRL) (*p *= 0.002). Endosialin expression in the tumour cells of both in the RT group (*p *= 0.01) and the non-RT group (*p *= 0.06) was observed more often in tumours with an infiltrative growth pattern than in tumours with an expansive growth pattern. In the RT group, endosialin expression in tumour cells was positively related to PRL expression (*p *= 0.02), whereas in the non-RT group, endosialin expression in tumour cells was positively related to p73 expression (*p *= 0.01).

**Conclusions:**

Endosialin expression may be involved in the progression of rectal cancers, and was related to Cox-2, p73 and PRL expression. However, a direct relationship between endosialin expression and RT responses in patients was not found.

## Background

Colorectal cancer is one of the most common malignant diseases in western countries. Rectal cancer is a frequent presentation, with an estimated 35% of cases found situated in the rectum [1]. New surgical techniques [2] and preoperative radiotherapy (RT) [3] have improved the local control and disease-free survival of patients with rectal cancer. However the incidence of recurrence and mortality are still high, even following RT. Therefore, it is important to gain a better understanding of the changes induced in tumours following RT of rectal cancer patients and search for new biological markers in order to evaluate their therapeutic effects.

The initiation and progression of tumours are influenced by the behaviour of the tumour microenvironment, consisting of the extracellular matrix (ECM), the newly formed vasculature, inflammatory cells and fibroblasts [4,5]. Tumour-associated fibroblasts (activated fibroblasts and myofibroblasts) have a well-recognized role as a source of paracrine (cell-to-cell) growth factors that influence the growth, migration and invasion of cancer cells during the carcinogenic process. Activated fibroblasts are also responsible for the synthesis, deposition and remodelling of the ECM in tumour-associated stroma [4]. Angiogenesis is a multistep process in tumour progression that involves both endothelial cells and pericytes. Alternative potential targets for inhibiting tumours may be involved in the tumour-associated stroma that contains newly formed blood vessels, active fibroblasts and ECM proteins.

One of these ECM proteins is endosialin. The gene for this protein is located in chromosome 11q13 [6], and its product is a type I transmembrane protein, which is a highly sialylated cell surface receptor found conserved in humans and in mice. Its extracellular portion consists of five globular domains, which are N-terminal C-type lectin domain, a sushi-like domain, and three epidermal growth factor (EGF)-like repeats, followed by a mucin-like region [7,8]. Endosialin was first reported to be selectively expressed in tumour-associated endothelium, which results in an alternate designation of tumour endothelial marker 1 (TEM1) [9]. Recently, this designation was challenged by a series of studies in which endosialin was shown to be expressed in pericytes (periendothelial mural cells) and activated fibroblasts [10-14]. Thus, endosialin plays an important role in overall tumour vasculature [15]. Targeting on endosialin or its related pathways may therefore offer an attractive therapeutic opportunity for cancer patients [12].

In the present study, we examined endosialin expression in distant and adjacent normal mucosa, as well as in primary tumours, from rectal cancer patients, with or without preoperative RT. We aimed to investigate the relationships of endosialin expression with RT responses, and clinicopathological and biological variables associated with rectal cancers.

## Methods

### Patients

Endosialin was immunohistochemically examined in distant mucosa samples (*n *= 72, in which 65 cases were matched with primary tumours), adjacent normal mucosa samples (*n *= 112) and primary tumours (*n *= 135) from the patients with rectal adenocarcinoma. The patients were from the Southeast Swedish Health Care region and participated in a Swedish clinical trial of preoperative RT between 1987 and 1990 [3]. The distant normal mucosal samples were taken from a resected distant margin that was histologically free from tumours, and adjacent normal mucosa was adjacent to the primary tumour on the same histologic section. The study was approved by the ethical committee of the Faculty of Health Sciences, Universities of Linköping and Uppsala, Sweden. All participants gave informed consents.

Among 135 patients, 73 patients received surgery alone and 62 received additional preoperative RT. A total of 25 Gy of radiation was administered in five fractions before surgery, over a median of 8 days (range, 6-15 days). Surgery was performed at a median of 3 days (range, 0-8 days) after RT. None of the patients received adjuvant chemotherapy before or after surgery. The mean age of the patients was 67 years (range, 36-85 years; median, 69 years). All patients were included in the follow-up, with mean and median follow-up periods of 86 and 75 months (range, 0-193 months), respectively. Follow-up sessions were scheduled at the end of 2004, by which time 49 patients had died from rectal cancer.

The growth pattern of the tumours was classified (by two pathologists) as either expansive or infiltrative pattern, based on their patterns of growth and invasiveness. Tumours were graded as well, moderately or poorly differentiated. Other patient and tumour characteristics are presented in Table 1. No statistically significant differences between the non-RT and RT groups regarding gender, age, TNM stage, grade of differentiation, and number of other tumours, surgical type, resection margin and mean distance to the anal verge were found (*p *> 0.05).

**Table 1:** Characteristics of patients and rectal cancers

Characteristics	Non-Radiotherapy	Radiotherapy	*p *-value
			
	n. (%)	n. (%)	
Gender			0.64
Male	43 (59)	39 (63)	
Female	30 (41)	23 (37)	
Age (years)			0.65
≤67	29 (40)	27 (44)	
>67	44 (60)	35 (56)	
TNM stage			0.26
I	21 (51)	20 (49)	
II	18 (47)	20 (53)	
III	29 (66)	15 (34)	
IV	5 (42)	7 (58)	
Differentiation			0.87
Well	2 (3)	2 (3)	
Moderately	57 (78)	46 (74)	
Poorly	14 (19)	14 (23)	
Numbers of other tumours*			0.07
Single	63 (86)	47 (76)	
Multiple	8 (11)	14 (22)	
Surgical type			0.16
Rectal amputation	37 (51)	24 (39)	
Anterior resection	36 (49)	38 (61)	
Resection margin			0.25
Tumour free	71 (97)	57 (92)	
Tumour	2 (3)	5 (8)	
To anal verge (cm)			0.14^#^
Mean	7.67	8.67	

* Other colorectal cancer and/or other type of tumour besides the present rectal cancer

^# ^For T-test, and the other *p *values for X^2 ^test

The data for expression of cyclooxygenase-2 (Cox-2) [16], p73 [17] and phosphates of regenerating liver (PRL) [18] determined by immunohistochemistry, were obtained from the previous studies carried out at our laboratory.

### Immunohistochemical staining

The mouse monoclonal antibody (B1/35), which is directed against human endosialin, was provided by Prof. Clare Isacke (Institute of Cancer Research, Sutton, UK), and described previously [11,12].

Five micrometer sections obtained from paraffin-embedded tissue blocks were incubated overnight at 60 °C, deparaffinized in xylene, and rehydrated in graded ethanol and distilled water. Sections were boiled in 0.01M citrate buffer (pH 6.0) in a high-pressure cooker for 1 min at 120 °C and then kept at room temperature for 30 min, followed by washing in phosphate-buffered saline (PBS; pH 7.4) buffer. The sections were then incubated overnight at 4°C with the primary antibody diluted to 1:250 in PBS. Sections were rinsed with PBS and incubated with polymerized horseradish peroxidise (HRP) -anti mouse/rabbit IgG for 30 min at room temperature (Real™ Envision™ HRP (rabbit/mouse) kit, Dako). After washing with PBS, the peroxidase reaction was run for 8 min with 3, 3'- diaminobenzididine (DAB). Sections were then counterstained with hematoxylin and mounted for microscopic examination.

The normal mucosa and primary tumour samples were stained in the same immunostaining run to avoid biases in the staining pattern and intensity. Sections known to stain positively were included as negative and positive controls. For negative controls, sections incubated with universal mouse IgG (Dako), instead of the primary antibody, were not stained, whereas, the positive controls were stained with the primary antibody.

The slides were examined with a microscope and scored independently by two pathologists who were given no clinical or pathological information. To avoid artifacts, areas with poor morphology, section margins, and any necrotic regions were not considered. The staining intensity in the entire stroma was scored as either weak (including negative) or strong. The staining intensity of tumour cells over the entire slide area was scored as negative or positive (if positive cells >5% of tumour cells).

### Statistical analysis

The significance of the difference in the intensity of endosialin expression between normal mucosa and primary tumours, as well as between stroma and tumour cells, was examined by X^2 ^or McNemar's test. The relationship between endosialin expression and clinicopathological/biological factors was examined by the X^2 ^method. The relationship between endosialin expression and survival was tested by using Cox's proportional hazard model. All *p *values were two sided, and values of p < 0.05 were considered as statistically significant.

## Results

### Endosialin expression in the stroma of distant normal mucosa, adjacent normal mucosa and tumour

**Figure 1:**
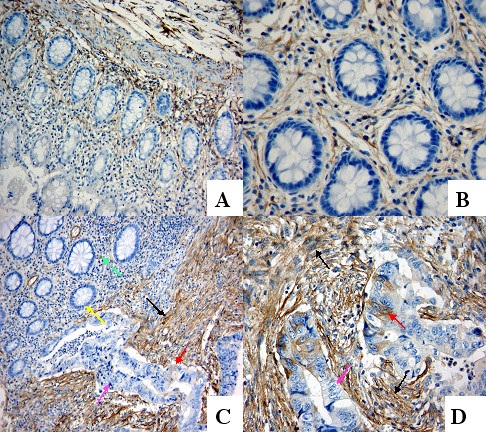
**Endosialin expression in rectal normal mucosa and in rectal cancer (representative images from different groups)**. (A and B) Weak expression in the fibroblasts and capillaries in the stroma, and lack of expression in epithelial cells of normal mucosa ((A) ×100 and (B) ×400). (C) Strong expression in fibroblasts of tumour-associated stroma (black arrow) and weak expression in fibroblasts of normal mucosa (green arrow), normal epithelial cells are negative (yellow arrow), and a few tumour cells are positive (red arrow) and negative (pink arrow) (×100). (D) Strong expression in fibroblasts of tumour-associated stroma (black arrow) and tumour cells (red arrow), whereas some tumour cells are negative (pink arrow) (×400).

Endosialin was expressed in fibroblasts and blood vessels in the stroma of normal mucosa, including distant and adjacent normal mucosa, and tumour (Figure 1A-D). In the fibroblasts, endosialin expression of intratumoural or peritumoural areas ranged from a prominent to a diffuse pattern. In the blood vessels (mainly mircrovessels), on the other hand, endosialin presented in the cells around vessels, either in the stroma of normal mucosa or tumour.

**Figure 2:**
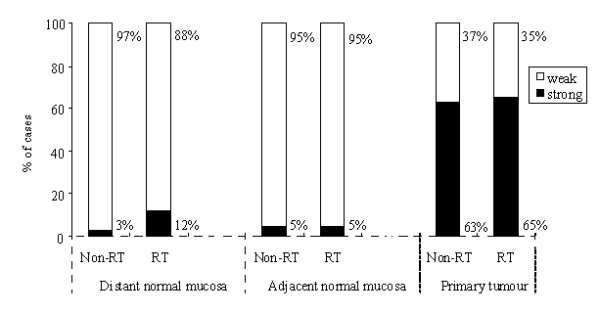
**Endosialin expression in the stroma in distant normal mucosa, adjacent normal mucosa and tumour in non-RT and RT groups**. A significant increase was observed from the distant normal mucosa or adjacent normal mucosa to the tumour (*p *< 0.0001).

Endosialin expression in the stroma significantly increased from distant or adjacent normal mucosa to the tumour (*p *< 0.0001) in both the non-RT group (3%, 5%, and 63%, respectively) and the RT groups (12%, 5%, and 65%, respectively, Figure 2). No significant difference was observed between distant and adjacent normal mucosa in the two subgroups (*p *> 0.05; Figure 2).

### Endosialin expression in the stroma in relation to clinicopathological and biological variables

As shown in Table 2, in the RT group, the frequencies of strong endosialin expression in the stroma in TNM stages I to IV were 45%, 70%, 87%, and 57%, respectively (*p *= 0.07). If TNM stage I (45%) was compared with other stages (II+III+IV, 74%) the difference was statistically significant (*p *= 0.03). Endosialin expression was positively associated with expression of Cox-2 (*p *= 0.03), p73 (*p *= 0.01), and PRL (*p *= 0.002), whereas there was no such relationship in the non-RT group (*p *> 0.05; Table 2). No significant associations of endosialin expression in the stroma with other clinicopathological variables, including gender, age, tumour location, differentiation, complication, local/distant recurrence, overall survival and disease-free survival, in either the RT group or the non-RT group (*p *> 0.05; data not shown) were also observed.

**Table 2:** Endosialin expression in tumour-associated stroma in relation to clinicopathological and biological variables in rectal cancer patients

Variables	Non-Radiotherapy		*p *-value	Radiotherapy		*p *-value
				
	Weak (%)	Strong (%)		Weak (%)	Strong (%)	
TNM stage			0.92			0.07
I	9 (43)	12 (57)		11 (55)	9 (45)	
II	6 (33)	12 (67)		6 (30)	14 (70)	
III	10 (34)	19 (66)		2 (13)	13 (87)	
IV	2 (40)	3 (60)		3 (43)	4 (57)	
Cox-2			0.89			0.03
Weak	14 (37)	24 (63)		13 (50)	13 (50)	
Strong	12 (35)	22 (65)		7 (22)	25 (78)	
p73			0.11			0.01
Weak	13 (46)	15 (54)		11 (55)	9 (45)	
Strong	12 (28)	31 (72)		9 (19)	26 (81)	
PRL			0.47			0.002
Weak	4 (44)	5 (56)		8 (73)	3 (27)	
Strong	18 (32)	38 (68)		10 (24)	32 (76)	

*p *values for X^2 ^test; Cox-2: cyclooxygenase-2; PRL: phosphates of regenerating liver.

### Endosialin expression in the epithelial cells of distant normal mucosa, adjacent normal mucosa and carcinoma cells of tumours

There was no positive expression in epithelial cells of distant and adjacent normal mucosa in either the non-RT group or the RT group (Figure 1A-C). While in 25 cases of non-RT group and 21 cases of RT group, some tumour cells had positive endosialin expression (Figure 1C-D).

We further analyzed the relationship between endosialin expression in the stroma and that in the tumour cells in non-RT and RT group (Table 3). The expression in the stroma and in the tumour cells was concordant in 24 weak/negative cases (33%) and 22 strong/positive cases (30%) in non-RT group. Only 27 cases (37%) showed different expression in the two locations (3 tumours (4%) showed positive expression in tumour cells but weak expression in stroma, and 24 tumours (33%) showed strong expression in stroma but negative expression in tumour cells). In the RT group, the concordance of endosialin expression in stroma and in tumour cells was 20 weak/negative cases (32%) and 19 strong/positive cases (31%). The discordance was 23 cases (37%), which showed different expression in the two locations (2 tumours (3%) showed positive expression in tumour cells but weak expression in stroma, and 21 tumours (34%) showed strong expression in stroma but negative expression in tumour cells). There was a significant difference between the expression in the stroma and in the tumour cells in the non-RT (*p *= 0.0001) and RT groups (*p *= 0.0003).

**Table 3:** Relationship of endosialin expression in stroma and tumour cells of rectal cancer patients

	Non-Radiotherapy			Radiotherapy		
				
Variables	Stroma Weak (%)	Stroma Strong (%)	*p-value	Stroma Weak (%)	Stroma Strong (%)	*p-value
Tumour cells			0.0001			0.0002
Negative (%)	24 (33)	24 (33)		20 (32)	21 (34)	
Positive (%)	3 (4)	22 (30)		2 (3)	19 (31)	
Total (%)	27 (37)	46 (63)		22 (35)	40 (65)	

* For McNemar's test.

### Endosialin expression in tumour cells in relation to clinicopathological and biological variables

In the non-RT group, endosialin expression was more frequent in tumours with infiltrative growth patterns than those with expansive growth patterns (*p *= 0.01; Table 4) and was positively related to p73 expression (*p *= 0.01; Table 4). In the RT group, a similar trend of endosialin expression was observed in tumours with infiltrative growth patterns when compared to those with expansive growth patterns (*p *= 0.06; Table 4). Endosialin expression was positively related to PRL expression (*p *= 0.02; Table 4).

**Table 4:** Endosialin expression in tumour cells in relation to clinicopathological and biological variables in rectal cancer patients

Variables	Non-Radiotherapy		*p*-value	Radiotherapy		*p*-value
				
	Negative (%)	Positive (%)		Negative (%)	Positive (%)	
Growth pattern			0.01			0.06
Expansive	35 (71)	14 (29)		21 (75)	7 (25)	
Infiltrative	7 (39)	11 (61)		12 (50)	12 (50)	
Cox-2			0.16			0.10
Weak	22 (58)	16 (42)		20 (77)	6 (23)	
Strong	25 (74)	9 (26)		18 (56)	14 (44)	
p73			0.01			0.44
Weak	23 (82)	5 (18)		14 (70)	6 (30)	
Strong	23 (53)	20 (47)		19 (59)	13 (41)	
PRL			0.25			0.02
Weak	14 (74)	5 (26)		16 (84)	3 (16)	
Strong	27 (59)	19 (41)		18 (53)	16 (47)	

*p *values for X^2 ^test; Cox-2: cyclooxygenase-2; PRL: phosphates of regenerating liver.

No significant correlation between endosialin expression in tumour cells and survival, recurrence and other pathological/biological variables were found in either the non-RT group or in the RT group (*p *>0.05; data not shown).

## Discussion

Endosialin, also referred to as TEM1, was originally discovered as a human embryonic fibroblast-specific antigen and later reported to be expressed in the endothelium. Endosialin is barely detectable in normal tissues other than its moderate expression in the smooth muscle of colon and prostate [13]. Therefore, TEM1 was once considered as an important candidate as a vascular target [6,9,19]. However, recent studies have demonstrated that endosialin is expressed in pericytes of breast tumours and brain gliomas, and not selectively in tumour endothelium [10-12]. In the present study, endosialin was prominently expressed in the tumour-associated stroma, especially in fibroblasts and blood vessels, and only weakly expressed in the stroma of distant or adjacent normal mucosa in both the non-RT group and in the RT groups. When compared to the normal mucosa, endosialin expression was much higher in tumour, thus agreeing with other reports on colon and breast cancers [20,21]. Up-regulated endosialin in the tumour-associated stroma may play a role in the tumorigenesis of rectal cancers.

In the present study, endosialin expression in the stroma was more frequently observed in advanced TNM stages, and positively related to the tumour cellular expression of Cox-2, p73, and PRL in the RT group, whereas there were no such associations in the non-RT group. The level of endosialin expression in the tumour-associated stroma was significantly higher in breast cancers with nodal involvement compared to those with negative nodes [21]. Endosialin has also been found in glioblastoma multiforme, anaplastic astrocytomas, and metastatic carcinomas that are of highly invasive activity [12,22]. Endosialin is also more abundant in melanoma metastases than in the primary tumours [13]. In colorectal cancer, endosialin was up-regulated in Dukes' B compared to Dukes' A [20]. Cox-2 is an inducible isoenzyme of cyclooxygenase that is undetectable in normal colonic mucosa but is overexpressed in 80% of colonic tumours [23]. Cox-2 is involved in a multistep process of colorectal tumorigenesis, such as apoptosis inhibition of cellular proliferation and angiogenesis enhancement, tumour cell invasion and differentiation. One of our previous studies has shown that Cox-2 expression is higher in more advanced tumours [24]. It is interesting to see, in the further study, whether or not endosialin and Cox-2 have interactions in the tumour development, especially in enhancing angiogenesis. We have also found that p73 expression is increased during the development of colorectal cancers and its overexpression is further associated with poor prognosis in patients [25]. PRL, which stimulates the Rho signalling pathway to promote cell motility and invasion [26], is up-regulated in colorectal cancer and associated with tumour invasion and metastasis [18]. In the same series of the patients, our previous studies have demonstrated that tumours with p53-negative expression (wild type p53), or p73-negative expression, or weak Cox-2 expression had less local recurrence after RT [16,17,27]. PRL expression is related to distant recurrence and poor survival after RT [18]. In further studies of colon cancer cell lines, we found that, after radiation, the antiapoptotic ΔNp73 and mitosis factor PRL-3 increase [28] and the overexpression of ΔNp73β increases the viability of cell lines and cisplatin induces the degradation of ΔNp73β in a dose-dependent manner (unpublished data). All these results indicate that certain biological factors may be involved in response to therapy in rectal cancer patients. If we could further confirm the data obtained from studies of these biological factors in relation to the clinicopathological issues above as well as their pathways in therapies, we may be able to apply them to clinical practices where rectal cancer patients may receive individual therapies based on their biological profile. For example, the targeting of multiple biological factors instead of only one in certain therapies may yield greater responses to them.

Radiated stromal fibroblasts in the carcinogenic process have been shown to induce sub-lethal DNA damage. Radiation-induced alterations of the stroma have been found to produce more mammary carcinomas compared to non-radiated stroma [29]. All of these results hinted that the patients with up-regulated expression of endosialin in a tumour-associated stroma may be linked to other biological variables that are related to aggressive characterization after radiation. However, we did not find that endosialin expression was further related to survival in either the whole group or subgroups (with or without RT) of patients. This may be partly due to the limited number of the patients and/or the hypothesis that endosialin may play a survival role in certain groups of the patients. It would be interesting to determine the survival significance of endosialin in subgroups where the endosialin is related to the clinicopathological variables, such as TNM, Cox-2, p73 and PRL. We did find that endosialin presented different results in each subgroup, but firm conclusions are difficult to draw at this point due to the limited number.

In the present study, we also observed endosialin expression in tumour cells. Positive endosialin expression in tumour cells was more frequently observed in tumours with infiltrative growth pattern compared to expansive growth pattern, regardless of RT. Endosialin was positively related to p73 expression in the non-RT group, and to PRL expression in the RT group. Why was stromal endosialin positively related to p73, PRL and Cox-2 in the RT-group, but not in the non-RT group? One speculation may be that radiation influenced biological factors by up-regulating or down-regulating expression. After radiation, "bad factors," such as Cox-2, p73, and PRL-3 increased their expression. This indicates that there may be some mechanism by which tumors try to protect themselves by increasing the expression of "bad factors" against the damage of radiation. Furthermore, why did stromal endosialin and tumour cellular endosialin have different relationships with biological factors (p73 and PRL)? For example, stromal endosialin was related to p73 expression in the RT group, whereas tumour cellular endosialin was related to p73 in the non-RT group. One possible explanation may be that the effects of radiation on stroma differ on different types of tumour cells, resulting in different expression and relationships to the biological factors investigated. In addition, the p73 gene contains two promoter regions, giving rise to a p53-like protein named TAp73, and the N-terminally truncated ΔNp73, which lacks the transactivation domain and p53 homology. ΔNp73 is thought to have a regulatory function, down-regulating both TAp73 by protein-protein interaction and p53 by competitive binding with DNA. In this autoregulatory loop, ΔNp73 protein is also up-regulated by both TAp73 and p53. The functional cooperation among these family members seems to vary depending on cell types, stimuli and p53 status [30]. In other words, the roles of the two isoforms may depend on their locations in the stroma or tumour cells.

Recent studies have raised the concept of the coevolution of tumour cells with tumour-associated stroma. The stromal environment of tumours appears to be a leading factor, and not just a supporting one in the initiation of tumours [31]. The tumour microenvironment and interactions between tumour and stromal cells have a reciprocal relationship in tumour development and progression. Another interesting concept is epithelial-mesenchymal transition (EMT), a process by which cells lose their polarized epithelial structures and concomitantly acquire a migratory or mesenchymal phenotype. EMT is essential for normal embryonic development and progression of non-invasive adenomas into malignant, metastatic carcinomas. Alterations in cell-cell adhesion, cell-substrate interaction, extracellular matrix degradation and cytoskeleton organization are the major events that occur during EMT [32]. Overexpressed thymosin β4 (Tβ4) induces EMT in colorectal carcinoma by increasing integrin-linked kinase (ILK) complex formation with particularly interesting new cysteine-histidine rich protein (PINCH) [32]. We have studied PINCH expression in colorectal carcinomas and found that PINCH overexpressed on fibroblasts in the tumour-associated stroma compared to its expression in normal mucosa [33], similar to endosialin expression in tumour versus normal mucosa. In the present study, endosialin was expressed in the both tumour-associated stroma and tumour cells. Furthermore, endosialin expression in the tumour-associated stroma was positively correlated with that in tumour cells, giving more information regarding the interactions of endosialin between tumour microenvironments and tumours. Changes in signal conduction related to endosialin appear to play an important role in enhancing tumour progression after radiation. There was evidence that antiangiogenic therapies targeting both endothelial cells and pericytes were more effective than single-agent therapies [34]. In the present study, endosialin was not only obviously up-regulated in stroma but also up-regulated in tumour cells when compared to normal mucosa. If the therapies target endosialin in both stroma and tumour cells, they may provide more efficient strategies of therapy for the patients with rectal cancers.

Regarding the discrepancies of endosialin localization, in stroma and/or in tumour cells found in different studies, several factors may be responsible, including the number and clinicopathological characteristics of the patients included in such studies, as well as the methods used in the same. For example, if tissue arrays were used for staining endosialin [13], it is possible that a limited sample of tissue was obtained from the tumour blocks and the selected arrays may not be representative of the complete characteristics of the tumour because of tumour heterogeneity. In the present study, we used ordinary sections for staining endosialin and observed that only 34% of the cases showed positive endosialin expression in tumour cells. In fact, in some positive cases, only a few positive tumour cells were determined in the entire tumour sections. In addition, some studies which employed real-time PCR or quantitative real-time PCR methods could not determine the location of the expression [20,21]. In the present study, we used immunohistochemical staining, which is one of the best methods of identifying the location of endosialin expression.

## Conclusions

Endosialin expression may be involved in the progression of rectal cancers. It is also related to Cox-2, p73, and PRL expression. However, a direct relationship between endosialin expression and RT responses in patients was not found.

## Competing interests

The authors declare that they have no competing interests.

## Authors' contributions

ZYZ conducted the experiments, analyzed the results with XFS, and wrote the drafts of the manuscript. ZYZ and HZ read the immunohistochemical slides. AG provided the samples for the experiments and information regarding the therapy. XFS designed the experiments and helped write the manuscript. All authors have read and approved the final manuscript.

## Pre-publication history

The pre-publication history for this paper can be accessed here:

http://www.biomedcentral.com/1471-2407/11/89/prepub
